# Supplementary material for: Splice-Junction-Based Mapping of Alternative Isoforms in the Human Proteome
Source: Cell Rep. Author manuscript; Available in PMC 2020 Jan 15. (PMC6961840; doi:10.1016/j.celrep.2019.11.026)
Supplement: 2 [file NIHMS1546469-supplement-2.zip › DF1/PXD010154/Thyroid/Thyroid_32_SRP14_AAAAAAAAAPAAAATAPTTAATTAATAAP.pdf]

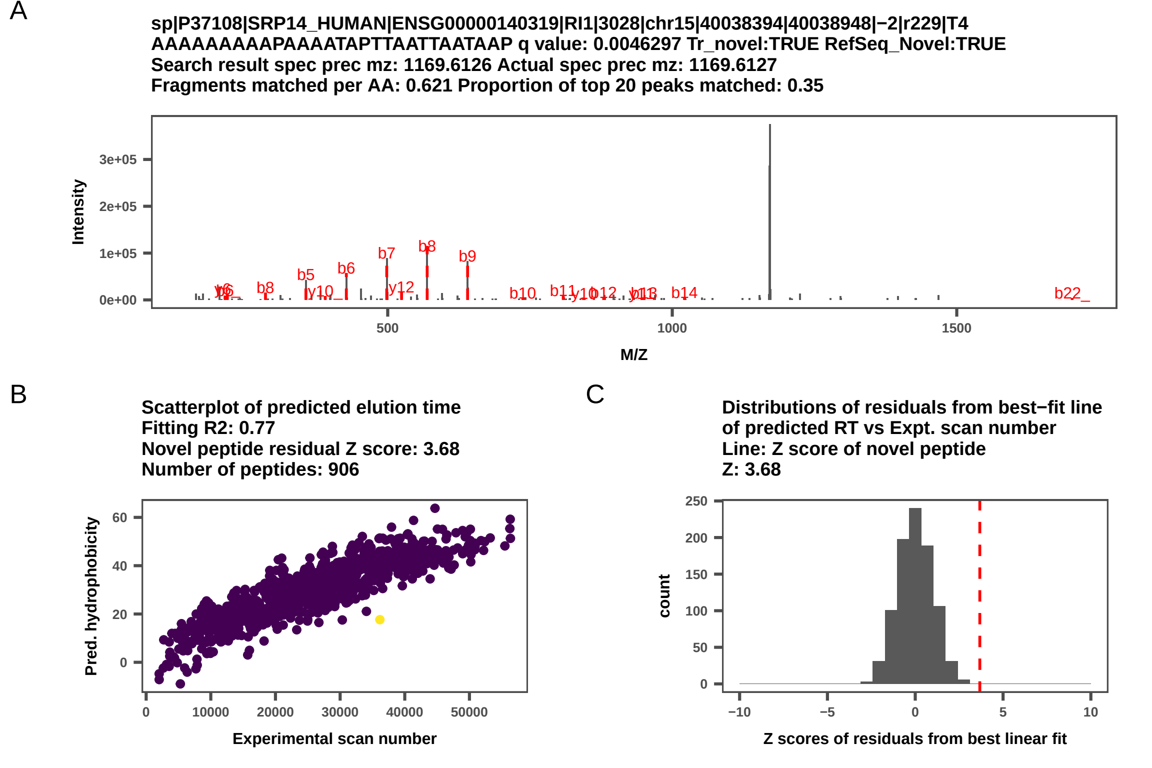
A
sp|P37108|SRP14_HUMAN|ENSG00000140319|RI1|3028|chr15|40038394|40038948|−2|r229|T4
AAAAAAAAAPAAAATAPTTAATTAATAAP q value: 0.0046297 Tr_novel:TRUE RefSeq_Novel:TRUE
Search result spec prec mz: 1169.6126 Actual spec prec mz: 1169.6127
Fragments matched per AA: 0.621 Proportion of top 20 peaks matched: 0.35
Intensity
3e+05
2e+05
1e+05
0e+00
b5
b6
b7
b8
b9
b8
y10
y12
b10
b11
b12
b13
b14
b22
500
1000
1500
M/Z
B
Scatterplot of predicted elution time
Fitting R2: 0.77
Novel peptide residual Z score: 3.68
Number of peptides: 906
Pred. hydrophobicity
60
40
20
0
0
10000
20000
30000
40000
50000
Experimental scan number
C
Distributions of residuals from best−fit line
of predicted RT vs Expt. scan number
Line: Z score of novel peptide
Z: 3.68
count
250
200
150
100
50
0
−10
−5
0
5
10
Z scores of residuals from best linear fit
